# (2,2′-Bipyridine-κ^2^ *N*,*N*′)chlorido[4′-(2,5-dimethoxy­phen­yl)-2,2′:6′,2′′-terpyridine-κ^3^ *N*,*N*′,*N*′′]ruthenium(II) hexa­fluorido­phosphate acetonitrile monosolvate

**DOI:** 10.1107/S1600536809032589

**Published:** 2009-08-22

**Authors:** Dai Oyama, Masato Kido, Ai Orita, Tsugiko Takase

**Affiliations:** a Department of Industrial Systems Engineering, Cluster of Science and Technology, Fukushima University, 1 Kanayagawa, Fukushima 960-1296, Japan; b Department of Science Education, Faculty of Education, Fukushima University, 1 Kanayagawa, Fukushima 960-1296, Japan

## Abstract

In the title compound, [RuCl(C_10_H_8_N_2_)(C_23_H_19_N_3_O_2_)]PF_6_·CH_3_CN, the ligand environment about the Ru^II^ atom is distorted octa­hedral, with the substituted terpyridyl ligand coordinated in a meridional fashion, the bipyridyl ligand coordinated in a *cis* fashion and the Cl atom *trans* to one of the bipyridyl N atoms. The Ru—N distances are in the range 2.036 (2)–2.084 (2) Å with the exception of the central Ru—N bond from the terpyridyl ligand, which is shorter [1.9503 (19) Å], as expected. The pendant dimethoxy­phenyl substituent is not coplanar with the terpyridyl unit; the dihedral angle between the central pyridyl ring and the benzene ring is 46.72 (11)°. The anion is disordered equally over two positions around an F—P—F bond axis.

## Related literature

For details of the synthesis, see: Takeuchi *et al.* (1984 ▶); Storrier *et al.* (1995 ▶, 1998 ▶). For related structures, see: Spek *et al.* (1994 ▶); Fujihara *et al.* (2003 ▶); Tseng *et al.* (2008 ▶). For general background to catalytic water oxidation using mononuclear ruthenium complexes, see: Tseng *et al.* (2008 ▶).
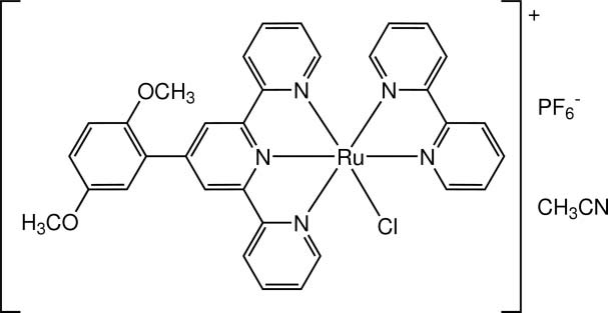


## Experimental

### 

#### Crystal data

[RuCl(C_10_H_8_N_2_)(C_23_H_19_N_3_O_2_)]PF_6_·C_2_H_3_N
*M* *_r_* = 848.15
Orthorhombic, 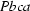

*a* = 13.8691 (3) Å
*b* = 16.1993 (3) Å
*c* = 31.5514 (6) Å
*V* = 7088.7 (2) Å^3^
*Z* = 8
Mo *K*α radiation
μ = 0.64 mm^−1^
*T* = 296 K
0.60 × 0.40 × 0.08 mm

#### Data collection

Rigaku R-AXIS RAPID diffractometer
Absorption correction: multi-scan (**ABSCOR**; Higashi, 1995 ▶) *T*_min_ = 0.616, *T*_max_ = 0.950
102710 measured reflections
8092 independent reflections
5738 reflections with *I* > 2σ(*I*)
*R*_int_ = 0.036

#### Refinement

*R*[*F* ^2^ > 2σ(*F* ^2^)] = 0.037
*wR*(*F* ^2^) = 0.111
*S* = 1.01
8092 reflections
495 parameters
All H-atom parameters refined
Δρ_max_ = 0.84 e Å^−3^
Δρ_min_ = −0.59 e Å^−3^

### 

Data collection: *PROCESS-AUTO* (Rigaku, 1998 ▶); cell refinement: *PROCESS-AUTO*; data reduction: *CrystalStructure* (Rigaku Americas & Rigaku, 2007 ▶); program(s) used to solve structure: *SIR97* (Altomare *et al.*, 1999 ▶); program(s) used to refine structure: *CRYSTALS* (Betteridge *et al.*, 2003 ▶); molecular graphics: *ORTEP-3 for Windows* (Farrugia, 1997 ▶); software used to prepare material for publication: *CrystalStructure*.

## Supplementary Material

Crystal structure: contains datablocks global, I. DOI: 10.1107/S1600536809032589/is2438sup1.cif

Structure factors: contains datablocks I. DOI: 10.1107/S1600536809032589/is2438Isup2.hkl

Additional supplementary materials:  crystallographic information; 3D view; checkCIF report

## Figures and Tables

**Table 1:** Selected bond lengths (Å)

Ru1—Cl1	2.4096 (8)
Ru1—N1	2.066 (2)
Ru1—N2	1.9503 (19)
Ru1—N3	2.082 (2)
Ru1—N4	2.036 (2)
Ru1—N5	2.084 (2)

**Fig. 1.**
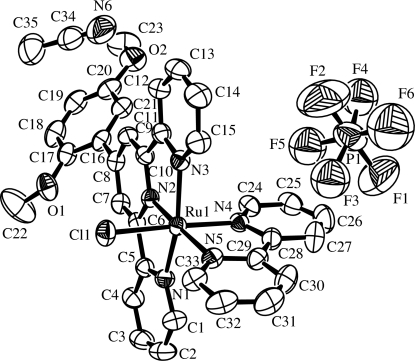
The molecular structure of the title compound, with atom labels and 50% probability displacement ellipsoids for non-H atoms. H atoms are omitted for clarity. Only one component of the disordered hexafluoridophosphate ion is shown.

## supplementary crystallographic information

### Comment

There have been numerous reports of ruthenium(II) polypyridyl complexes. In particular, a series of mononuclear ruthenium(II) complexes with both 2,2':6',2''-terpyridine (tpy) and bidentate pyridyl ligands (NN) has exhibited the catalytic activity toward water oxidation (Tseng *et al.*, 2008). We newly investigated the synthesis of ruthenium complexes bearing the substituted terpyridyl ligand because the absorption energies of the MLCT bands and the redox potentials of the complexes described above were consistent with their structures.

The ligand environment about the Ru atom is distorted octahedral, with the substituted terpyridyl ligand coordinated in a meridional fashion, the bipyridyl ligand coordinated in a *cis* fashion and the Cl atom *trans* to one of the bipyridyl N atoms (Fig. 1). The Ru—N distances are in the range of 2.036 (2)–2.084 (2) Å with the exception of the central Ru—N bond of the terpyridyl ligand, which is shorter [1.9503 (19) Å] as expected (Table 1). The Ru—Cl distance of 2.4096 (8) Å observed in this structure is similar to those found in other ruthenium(II)-terpyridine- chlorido complexes (Spek *et al.*, 1994; Fujihara *et al.*, 2003; Tseng *et al.*, 2008). The pendant dimethoxyphenyl substituent is not coplanar with the terpyridyl moiety; the dihedral angle between the central pyridyl and the dimethoxyphenyl ring is 46.72 (11)°. This result is essentially comparable to that found for the free ligand (50.2°) (Storrier *et al.*, 1998).

### Experimental

The ligand 4'-(2,5-dimethoxyphenyl)-2,2':6',2''-terpyridine (tpyOMe) was prepared and purified as described by Storrier *et al.* (1995 and 1998). The title compound was prepared following a procedure similar to that for the synthesis of [RuCl(bpy)(tpy)]PF_6_ (bpy = 2,2'-bipyridine, tpy = 2,2':6',2''-terpyridine) (Takeuchi *et al.*, 1984). X-ray quality crystals were grown by the diffusion of diethyl ether into an acetonitrile solution of the complex over a week.

### Refinement

Aromatic H atoms were fixed at C—H distances of 0.95 Å and refined as riding, with *U*_iso_(H) = 1.2*U*_eq_(C). Methyl H atoms were placed with idealized threefold symmetry and fixed C—H distances of 0.98 Å, and they were refined in a riding model with *U*_iso_(H) = 1.5*U*_eq_(C). Four F atoms in equatorial positions of the counter anion are disordered and were refined with the occupancy of all atoms fixed at 0.5.

### Crystal data

**Table:**

[RuCl(C_10_H_8_N_2_)(C_23_H_19_N_3_O_2_)]PF_6_·C_2_H_3_N	*F*(000) = 3424.00
*M**_r_* = 848.15	*D*_x_ = 1.589 Mg m^−^^3^
Orthorhombic, *P**b**c**a*	Mo *K*α radiation, λ = 0.71075 Å
Hall symbol: -P 2ac 2ab	Cell parameters from 82995 reflections
*a* = 13.8691 (3) Å	θ = 3.0–27.5°
*b* = 16.1993 (3) Å	µ = 0.64 mm^−^^1^
*c* = 31.5514 (6) Å	*T* = 296 K
*V* = 7088.7 (2) Å^3^	Platelet, black
*Z* = 8	0.60 × 0.40 × 0.08 mm

### Data collection

**Table:**

Rigaku R-AXIS RAPID diffractometer	5738 reflections with *F*^2^ > 2σ(*F*^2^)
Detector resolution: 10.00 pixels mm^-1^	*R*_int_ = 0.036
ω scans	θ_max_ = 27.5°
Absorption correction: multi-scan (*ABSCOR*; Higashi, 1995)	*h* = −17→17
*T*_min_ = 0.616, *T*_max_ = 0.950	*k* = −21→20
102710 measured reflections	*l* = −40→40
8092 independent reflections	

### Refinement

**Table:**

Refinement on *F*^2^	All H-atom parameters refined
*R*[*F*^2^ > 2σ(*F*^2^)] = 0.037	*w* = 1/[0.0008*F*_o_^2^ + σ(*F*_o_^2^)]/(4*F*_o_^2^)
*wR*(*F*^2^) = 0.111	(Δ/σ)_max_ < 0.001
*S* = 1.01	Δρ_max_ = 0.84 e Å^−^^3^
8092 reflections	Δρ_min_ = −0.59 e Å^−^^3^
495 parameters	

### Special details

**Table:**

Refinement. Refinement was performed using all reflections. The weighted *R*-factor (*wR*) and goodness of fit (*S*) are based on *F*^2^. *R*-factor (gt) are based on *F*. The threshold expression of *F*^2^ > 2.0 σ(*F*^2^) is used only for calculating *R*-factor (gt).

### Fractional atomic coordinates and isotropic or equivalent isotropic displacement parameters (Å^2^)

**Table:**

	*x*	*y*	*z*	*U*_iso_*/*U*_eq_	Occ. (<1)
Ru1	0.09533 (2)	0.785150 (10)	0.691700 (10)	0.03190 (6)	
Cl1	−0.07112 (6)	0.78760 (5)	0.71355 (2)	0.0516 (2)	
P1	0.28023 (11)	0.55855 (8)	0.55490 (4)	0.0907 (4)	
F1	0.3430 (3)	0.6325 (2)	0.53818 (13)	0.1835 (19)	
F2	0.2173 (4)	0.4867 (2)	0.56854 (18)	0.211 (2)	
F3	0.1973 (5)	0.6208 (4)	0.5418 (2)	0.135 (2)*	0.50
F4	0.3566 (5)	0.5038 (4)	0.5779 (2)	0.127 (2)*	0.50
F5	0.2666 (6)	0.6000 (4)	0.6016 (2)	0.132 (2)*	0.50
F6	0.2894 (9)	0.5187 (6)	0.5111 (3)	0.195 (3)*	0.50
F7	0.2059 (6)	0.6174 (5)	0.5747 (3)	0.163 (3)*	0.50
F8	0.3705 (5)	0.5057 (4)	0.5349 (2)	0.133 (2)*	0.50
F9	0.3448 (7)	0.5796 (5)	0.5938 (2)	0.160 (2)*	0.50
F10	0.2239 (7)	0.5570 (6)	0.5108 (2)	0.167 (3)*	0.50
O1	0.12400 (18)	0.83112 (16)	0.90412 (7)	0.0633 (7)	
O2	0.41159 (19)	0.59908 (16)	0.92417 (8)	0.0699 (8)	
N1	0.11237 (17)	0.90625 (13)	0.71081 (7)	0.0375 (6)	
N2	0.13304 (17)	0.76860 (12)	0.75064 (6)	0.0336 (5)	
N3	0.09369 (16)	0.65680 (14)	0.69479 (6)	0.0357 (5)	
N4	0.23041 (16)	0.78277 (12)	0.66643 (7)	0.0358 (5)	
N5	0.06613 (18)	0.80557 (13)	0.62776 (7)	0.0389 (6)	
N6	0.1768 (3)	0.4872 (2)	0.86305 (15)	0.1152 (17)	
C1	0.1022 (2)	0.97542 (19)	0.68752 (10)	0.0490 (8)	
C2	0.1122 (2)	1.0527 (2)	0.70476 (12)	0.0591 (10)	
C3	0.1352 (2)	1.06143 (18)	0.74710 (11)	0.0550 (9)	
C4	0.1486 (2)	0.99131 (17)	0.77096 (10)	0.0461 (8)	
C5	0.1378 (2)	0.91387 (16)	0.75254 (8)	0.0379 (7)	
C6	0.15085 (19)	0.83516 (15)	0.77544 (8)	0.0347 (6)	
C7	0.1817 (2)	0.82465 (17)	0.81662 (8)	0.0392 (7)	
C8	0.1916 (2)	0.74570 (17)	0.83322 (8)	0.0383 (7)	
C9	0.1693 (2)	0.67785 (17)	0.80744 (8)	0.0388 (7)	
C10	0.1395 (2)	0.69120 (15)	0.76611 (8)	0.0347 (6)	
C11	0.1159 (2)	0.62689 (16)	0.73418 (8)	0.0374 (7)	
C12	0.1172 (2)	0.54346 (17)	0.74277 (9)	0.0444 (8)	
C13	0.0981 (2)	0.48827 (19)	0.71038 (11)	0.0553 (9)	
C14	0.0772 (2)	0.5174 (2)	0.67092 (11)	0.0609 (10)	
C15	0.0751 (2)	0.60201 (18)	0.66405 (9)	0.0510 (9)	
C16	0.2300 (2)	0.73057 (17)	0.87661 (8)	0.0416 (7)	
C17	0.1949 (2)	0.77434 (19)	0.91198 (9)	0.0476 (8)	
C18	0.2308 (2)	0.7558 (2)	0.95182 (10)	0.0613 (10)	
C19	0.3015 (2)	0.6975 (2)	0.95717 (10)	0.0602 (10)	
C20	0.3381 (2)	0.6556 (2)	0.92280 (10)	0.0534 (9)	
C21	0.3013 (2)	0.67203 (18)	0.88303 (9)	0.0461 (8)	
C22	0.0980 (4)	0.8867 (3)	0.93613 (16)	0.125 (2)	
C23	0.4600 (3)	0.5899 (2)	0.96367 (13)	0.0905 (15)	
C24	0.3119 (2)	0.77412 (17)	0.68875 (10)	0.0448 (8)	
C25	0.4017 (2)	0.77157 (19)	0.67025 (13)	0.0561 (10)	
C26	0.4093 (2)	0.7782 (2)	0.62678 (15)	0.0699 (12)	
C27	0.3254 (2)	0.7889 (2)	0.60369 (12)	0.0678 (11)	
C28	0.2365 (2)	0.79113 (17)	0.62360 (9)	0.0436 (7)	
C29	0.1442 (2)	0.80328 (17)	0.60169 (9)	0.0427 (7)	
C30	0.1343 (3)	0.8150 (2)	0.55851 (10)	0.0622 (10)	
C31	0.0462 (3)	0.8315 (2)	0.54151 (10)	0.0658 (11)	
C32	−0.0323 (2)	0.8366 (2)	0.56786 (10)	0.0592 (10)	
C33	−0.0201 (2)	0.82218 (18)	0.61080 (9)	0.0475 (8)	
C34	0.1426 (3)	0.5146 (2)	0.89184 (15)	0.0766 (13)	
C35	0.0968 (3)	0.5501 (3)	0.92876 (18)	0.110 (2)	
H1	0.0874	0.9705	0.6582	0.058*	
H2	0.1031	1.1003	0.6876	0.071*	
H3	0.1417	1.1145	0.7596	0.066*	
H4	0.1654	0.9957	0.8001	0.055*	
H5	0.1950	0.8714	0.8338	0.047*	
H6	0.1746	0.6232	0.8182	0.046*	
H7	0.1313	0.5241	0.7705	0.053*	
H8	0.0983	0.4306	0.7157	0.066*	
H9	0.0657	0.4804	0.6481	0.073*	
H10	0.0596	0.6220	0.6366	0.061*	
H11	0.2061	0.7844	0.9758	0.074*	
H12	0.3250	0.6857	0.9848	0.072*	
H13	0.3254	0.6426	0.8592	0.055*	
H14	0.0702	0.9360	0.9229	0.150*	
H15	0.0504	0.8615	0.9551	0.150*	
H16	0.1554	0.9022	0.9524	0.150*	
H17	0.4645	0.6440	0.9775	0.109*	
H18	0.4241	0.5518	0.9819	0.109*	
H19	0.5250	0.5682	0.9588	0.109*	
H20	0.3077	0.7693	0.7187	0.054*	
H21	0.4582	0.7652	0.6870	0.067*	
H22	0.4702	0.7757	0.6130	0.083*	
H23	0.3292	0.7949	0.5738	0.082*	
H24	0.1893	0.8120	0.5407	0.075*	
H25	0.0388	0.8399	0.5119	0.079*	
H26	−0.0943	0.8494	0.5568	0.071*	
H27	−0.0749	0.8244	0.6288	0.057*	
H28	0.1419	0.5893	0.9417	0.132*	
H29	0.0809	0.5067	0.9492	0.132*	
H30	0.0378	0.5792	0.9205	0.132*	

### Atomic displacement parameters (Å^2^)

**Table:**

	*U*^11^	*U*^22^	*U*^33^	*U*^12^	*U*^13^	*U*^23^
Ru1	0.03289 (13)	0.03422 (12)	0.02858 (12)	−0.00031 (9)	−0.00081 (8)	0.00003 (8)
Cl1	0.0380 (4)	0.0673 (4)	0.0496 (4)	0.0000 (3)	0.0067 (3)	0.0027 (3)
P1	0.1047 (10)	0.0843 (8)	0.0833 (7)	0.0299 (7)	−0.0148 (7)	0.0013 (6)
F1	0.217 (4)	0.135 (3)	0.198 (4)	−0.048 (3)	0.063 (3)	0.027 (2)
F2	0.232 (5)	0.116 (2)	0.286 (6)	−0.057 (3)	0.019 (4)	0.040 (3)
O1	0.0577 (15)	0.0826 (17)	0.0495 (12)	0.0089 (13)	0.0019 (11)	−0.0175 (11)
O2	0.0721 (18)	0.0796 (17)	0.0579 (14)	0.0122 (13)	−0.0194 (12)	0.0128 (12)
N1	0.0372 (13)	0.0350 (11)	0.0403 (12)	0.0010 (9)	−0.0004 (9)	0.0022 (9)
N2	0.0359 (12)	0.0349 (10)	0.0300 (10)	0.0001 (9)	0.0014 (9)	−0.0006 (8)
N3	0.0386 (13)	0.0371 (11)	0.0316 (10)	−0.0018 (9)	−0.0006 (9)	−0.0034 (8)
N4	0.0345 (12)	0.0326 (10)	0.0404 (11)	−0.0010 (9)	0.0006 (9)	−0.0029 (9)
N5	0.0438 (14)	0.0383 (11)	0.0346 (11)	−0.0040 (10)	−0.0026 (10)	0.0008 (9)
N6	0.164 (4)	0.073 (2)	0.109 (3)	−0.024 (2)	0.049 (3)	−0.009 (2)
C1	0.057 (2)	0.0413 (15)	0.0482 (16)	−0.0014 (14)	−0.0045 (14)	0.0072 (12)
C2	0.063 (2)	0.0361 (15)	0.078 (2)	0.0009 (15)	−0.0082 (18)	0.0108 (15)
C3	0.052 (2)	0.0349 (14)	0.078 (2)	−0.0009 (14)	−0.0027 (18)	−0.0034 (14)
C4	0.0459 (18)	0.0400 (14)	0.0526 (16)	−0.0036 (13)	−0.0030 (14)	−0.0071 (12)
C5	0.0345 (15)	0.0370 (13)	0.0420 (14)	−0.0005 (11)	0.0022 (12)	−0.0015 (11)
C6	0.0311 (14)	0.0362 (13)	0.0368 (12)	−0.0002 (11)	0.0030 (10)	−0.0050 (10)
C7	0.0389 (16)	0.0429 (14)	0.0356 (13)	−0.0045 (12)	−0.0004 (11)	−0.0062 (11)
C8	0.0342 (15)	0.0467 (14)	0.0341 (13)	−0.0025 (12)	0.0007 (11)	−0.0013 (11)
C9	0.0410 (16)	0.0392 (14)	0.0362 (13)	0.0002 (12)	−0.0024 (11)	0.0029 (11)
C10	0.0337 (14)	0.0344 (12)	0.0361 (12)	0.0005 (11)	−0.0004 (11)	−0.0029 (10)
C11	0.0370 (16)	0.0389 (14)	0.0364 (13)	−0.0001 (11)	0.0003 (11)	−0.0037 (11)
C12	0.0468 (18)	0.0384 (13)	0.0480 (16)	0.0015 (13)	−0.0011 (13)	−0.0006 (12)
C13	0.064 (2)	0.0365 (15)	0.065 (2)	0.0006 (14)	−0.0008 (17)	−0.0074 (14)
C14	0.081 (2)	0.0491 (18)	0.0523 (18)	−0.0067 (17)	−0.0028 (18)	−0.0184 (15)
C15	0.066 (2)	0.0468 (16)	0.0400 (15)	−0.0034 (15)	−0.0049 (14)	−0.0083 (12)
C16	0.0433 (17)	0.0496 (16)	0.0318 (12)	−0.0108 (13)	−0.0016 (11)	0.0009 (11)
C17	0.0477 (18)	0.0584 (18)	0.0368 (14)	−0.0078 (15)	0.0009 (12)	−0.0044 (12)
C18	0.068 (2)	0.083 (2)	0.0324 (14)	−0.018 (2)	0.0028 (15)	−0.0043 (15)
C19	0.069 (2)	0.077 (2)	0.0344 (15)	−0.0134 (19)	−0.0092 (15)	0.0111 (14)
C20	0.059 (2)	0.0560 (18)	0.0455 (16)	−0.0121 (16)	−0.0117 (14)	0.0101 (14)
C21	0.0506 (19)	0.0511 (16)	0.0365 (14)	−0.0068 (14)	−0.0055 (12)	0.0026 (12)
C22	0.154 (5)	0.132 (4)	0.090 (3)	0.062 (3)	−0.023 (3)	−0.053 (3)
C23	0.104 (3)	0.090 (2)	0.077 (2)	0.011 (2)	−0.047 (2)	0.017 (2)
C24	0.0373 (16)	0.0424 (15)	0.0546 (17)	0.0003 (12)	−0.0027 (13)	−0.0055 (12)
C25	0.0349 (17)	0.0505 (18)	0.083 (2)	−0.0001 (13)	−0.0053 (16)	−0.0052 (16)
C26	0.041 (2)	0.078 (2)	0.091 (2)	−0.0019 (17)	0.0197 (19)	−0.008 (2)
C27	0.058 (2)	0.091 (2)	0.055 (2)	−0.006 (2)	0.0189 (17)	−0.0058 (18)
C28	0.0441 (17)	0.0442 (15)	0.0425 (14)	−0.0023 (13)	0.0073 (12)	−0.0029 (12)
C29	0.0496 (18)	0.0418 (15)	0.0369 (13)	−0.0055 (13)	0.0016 (12)	−0.0018 (11)
C30	0.073 (2)	0.079 (2)	0.0346 (15)	−0.010 (2)	0.0076 (16)	−0.0001 (15)
C31	0.087 (2)	0.073 (2)	0.0366 (15)	−0.011 (2)	−0.0155 (18)	0.0067 (15)
C32	0.071 (2)	0.0543 (18)	0.0524 (17)	−0.0112 (17)	−0.0247 (17)	0.0096 (14)
C33	0.0445 (18)	0.0510 (16)	0.0470 (15)	−0.0059 (14)	−0.0091 (13)	0.0050 (13)
C34	0.091 (3)	0.058 (2)	0.081 (2)	−0.014 (2)	0.011 (2)	0.003 (2)
C35	0.112 (4)	0.089 (3)	0.128 (4)	−0.028 (2)	0.042 (3)	−0.023 (3)

### Geometric parameters (Å, °)

**Table:**

Ru1—Cl1	2.4096 (8)	C16—C17	1.409 (3)
Ru1—N1	2.066 (2)	C16—C21	1.385 (4)
Ru1—N2	1.9503 (19)	C17—C18	1.385 (4)
Ru1—N3	2.082 (2)	C18—C19	1.371 (5)
Ru1—N4	2.036 (2)	C19—C20	1.376 (4)
Ru1—N5	2.084 (2)	C20—C21	1.380 (4)
P1—F1	1.573 (4)	C24—C25	1.376 (4)
P1—F2	1.517 (4)	C25—C26	1.380 (6)
P1—F3	1.585 (7)	C26—C27	1.383 (5)
P1—F4	1.560 (7)	C27—C28	1.384 (5)
P1—F5	1.631 (7)	C28—C29	1.468 (4)
P1—F6	1.529 (10)	C29—C30	1.383 (4)
P1—F7	1.537 (9)	C30—C31	1.360 (5)
P1—F8	1.642 (7)	C31—C32	1.373 (5)
P1—F9	1.557 (9)	C32—C33	1.385 (4)
P1—F10	1.596 (9)	C34—C35	1.447 (7)
O1—C17	1.369 (4)	C1—H1	0.950
O1—C22	1.400 (5)	C2—H2	0.950
O2—C20	1.371 (4)	C3—H3	0.950
O2—C23	1.423 (5)	C4—H4	0.950
N1—C1	1.347 (3)	C7—H5	0.950
N1—C5	1.369 (3)	C9—H6	0.950
N2—C6	1.355 (3)	C12—H7	0.950
N2—C10	1.348 (3)	C13—H8	0.950
N3—C11	1.369 (3)	C14—H9	0.950
N3—C15	1.340 (3)	C15—H10	0.950
N4—C24	1.339 (3)	C18—H11	0.950
N4—C28	1.361 (3)	C19—H12	0.950
N5—C29	1.361 (3)	C21—H13	0.950
N5—C33	1.337 (3)	C22—H14	0.980
N6—C34	1.116 (6)	C22—H15	0.980
C1—C2	1.372 (4)	C22—H16	0.980
C2—C3	1.381 (5)	C23—H17	0.980
C3—C4	1.375 (4)	C23—H18	0.980
C4—C5	1.391 (3)	C23—H19	0.980
C5—C6	1.477 (3)	C24—H20	0.950
C6—C7	1.378 (3)	C25—H21	0.950
C7—C8	1.389 (3)	C26—H22	0.950
C8—C9	1.402 (3)	C27—H23	0.950
C8—C16	1.489 (3)	C30—H24	0.950
C9—C10	1.385 (3)	C31—H25	0.950
C10—C11	1.486 (3)	C32—H26	0.950
C11—C12	1.379 (3)	C33—H27	0.950
C12—C13	1.384 (4)	C35—H28	0.980
C13—C14	1.363 (4)	C35—H29	0.980
C14—C15	1.387 (4)	C35—H30	0.980
			
Cl1—Ru1—N1	90.60 (6)	C8—C16—C21	120.1 (2)
Cl1—Ru1—N2	89.22 (7)	C17—C16—C21	118.4 (2)
Cl1—Ru1—N3	89.58 (6)	O1—C17—C16	116.3 (2)
Cl1—Ru1—N4	173.57 (6)	O1—C17—C18	124.7 (2)
Cl1—Ru1—N5	95.06 (7)	C16—C17—C18	119.0 (2)
N1—Ru1—N2	79.72 (8)	C17—C18—C19	121.2 (3)
N1—Ru1—N3	159.40 (8)	C18—C19—C20	120.4 (3)
N1—Ru1—N4	91.55 (8)	O2—C20—C19	125.4 (3)
N1—Ru1—N5	98.86 (8)	O2—C20—C21	115.6 (2)
N2—Ru1—N3	79.69 (7)	C19—C20—C21	119.0 (3)
N2—Ru1—N4	97.12 (9)	C16—C21—C20	121.9 (2)
N2—Ru1—N5	175.52 (9)	N4—C24—C25	123.0 (3)
N3—Ru1—N4	90.54 (8)	C24—C25—C26	119.2 (3)
N3—Ru1—N5	101.64 (7)	C25—C26—C27	118.0 (3)
N4—Ru1—N5	78.63 (9)	C26—C27—C28	120.9 (3)
F1—P1—F2	176.8 (2)	N4—C28—C27	120.2 (2)
F1—P1—F3	80.2 (3)	N4—C28—C29	115.3 (2)
F1—P1—F4	102.3 (3)	C27—C28—C29	124.5 (2)
F1—P1—F5	93.1 (3)	N5—C29—C28	114.4 (2)
F1—P1—F6	88.5 (4)	N5—C29—C30	120.8 (3)
F1—P1—F7	92.0 (3)	C28—C29—C30	124.8 (3)
F1—P1—F8	81.1 (3)	C29—C30—C31	120.4 (3)
F1—P1—F9	77.2 (3)	C30—C31—C32	119.0 (3)
F1—P1—F10	89.5 (3)	C31—C32—C33	119.0 (3)
F2—P1—F3	98.3 (3)	N5—C33—C32	122.4 (2)
F2—P1—F4	79.8 (3)	N6—C34—C35	179.0 (5)
F2—P1—F5	89.6 (3)	N1—C1—H1	118.9
F2—P1—F6	88.8 (4)	C2—C1—H1	119.0
F2—P1—F7	88.6 (3)	C1—C2—H2	120.2
F2—P1—F8	98.5 (3)	C3—C2—H2	119.8
F2—P1—F9	106.0 (4)	C2—C3—H3	121.1
F2—P1—F10	87.4 (4)	C4—C3—H3	120.5
F3—P1—F4	167.4 (4)	C3—C4—H4	120.1
F3—P1—F5	83.7 (4)	C5—C4—H4	119.8
F3—P1—F6	95.3 (5)	C6—C7—H5	120.0
F4—P1—F5	83.8 (3)	C8—C7—H5	120.0
F4—P1—F6	97.1 (5)	C8—C9—H6	120.4
F5—P1—F6	178.0 (5)	C10—C9—H6	120.3
F7—P1—F8	172.1 (4)	C11—C12—H7	120.5
F7—P1—F9	85.9 (5)	C13—C12—H7	120.5
F7—P1—F10	92.1 (5)	C12—C13—H8	120.3
F8—P1—F9	88.8 (4)	C14—C13—H8	120.3
F8—P1—F10	91.7 (4)	C13—C14—H9	120.6
F9—P1—F10	166.4 (4)	C15—C14—H9	120.0
C17—O1—C22	119.1 (3)	N3—C15—H10	118.6
C20—O2—C23	116.6 (2)	C14—C15—H10	119.0
Ru1—N1—C1	128.21 (19)	C17—C18—H11	119.1
Ru1—N1—C5	113.32 (16)	C19—C18—H11	119.7
C1—N1—C5	118.5 (2)	C18—C19—H12	119.8
Ru1—N2—C6	119.35 (16)	C20—C19—H12	119.8
Ru1—N2—C10	119.39 (16)	C16—C21—H13	118.7
C6—N2—C10	121.2 (2)	C20—C21—H13	119.4
Ru1—N3—C11	113.17 (16)	O1—C22—H14	108.6
Ru1—N3—C15	129.04 (17)	O1—C22—H15	110.4
C11—N3—C15	117.8 (2)	O1—C22—H16	109.5
Ru1—N4—C24	124.92 (19)	H14—C22—H15	109.5
Ru1—N4—C28	116.36 (18)	H14—C22—H16	109.5
C24—N4—C28	118.7 (2)	H15—C22—H16	109.5
Ru1—N5—C29	115.22 (19)	O2—C23—H17	109.0
Ru1—N5—C33	126.37 (19)	O2—C23—H18	110.0
C29—N5—C33	118.4 (2)	O2—C23—H19	109.5
N1—C1—C2	122.1 (2)	H17—C23—H18	109.5
C1—C2—C3	120.0 (3)	H17—C23—H19	109.5
C2—C3—C4	118.4 (2)	H18—C23—H19	109.5
C3—C4—C5	120.1 (2)	N4—C24—H20	118.7
N1—C5—C4	120.7 (2)	C25—C24—H20	118.4
N1—C5—C6	115.1 (2)	C24—C25—H21	120.9
C4—C5—C6	124.1 (2)	C26—C25—H21	119.9
N2—C6—C5	112.5 (2)	C25—C26—H22	121.2
N2—C6—C7	120.2 (2)	C27—C26—H22	120.8
C5—C6—C7	127.3 (2)	C26—C27—H23	119.3
C6—C7—C8	120.0 (2)	C28—C27—H23	119.8
C7—C8—C9	118.8 (2)	C29—C30—H24	119.8
C7—C8—C16	122.3 (2)	C31—C30—H24	119.9
C9—C8—C16	118.9 (2)	C30—C31—H25	120.9
C8—C9—C10	119.3 (2)	C32—C31—H25	120.1
N2—C10—C9	120.4 (2)	C31—C32—H26	120.5
N2—C10—C11	113.1 (2)	C33—C32—H26	120.5
C9—C10—C11	126.5 (2)	N5—C33—H27	118.9
N3—C11—C10	114.6 (2)	C32—C33—H27	118.7
N3—C11—C12	121.9 (2)	C34—C35—H28	108.3
C10—C11—C12	123.5 (2)	C34—C35—H29	110.0
C11—C12—C13	119.1 (2)	C34—C35—H30	110.1
C12—C13—C14	119.4 (2)	H28—C35—H29	109.5
C13—C14—C15	119.3 (3)	H28—C35—H30	109.5
N3—C15—C14	122.5 (2)	H29—C35—H30	109.5
C8—C16—C17	121.5 (2)		
			
Cl1—Ru1—N1—C1	−92.6 (2)	Ru1—N4—C28—C27	−179.0 (2)
Cl1—Ru1—N1—C5	87.90 (18)	Ru1—N4—C28—C29	1.8 (2)
Cl1—Ru1—N2—C6	−90.20 (19)	C24—N4—C28—C27	1.3 (3)
Cl1—Ru1—N2—C10	88.8 (2)	C24—N4—C28—C29	−177.9 (2)
Cl1—Ru1—N3—C11	−87.82 (17)	C28—N4—C24—C25	−1.4 (3)
Cl1—Ru1—N3—C15	92.8 (2)	Ru1—N5—C29—C28	−3.1 (2)
Cl1—Ru1—N5—C29	−175.68 (17)	Ru1—N5—C29—C30	179.3 (2)
Cl1—Ru1—N5—C33	6.0 (2)	Ru1—N5—C33—C32	178.7 (2)
N1—Ru1—N2—C6	0.6 (2)	C29—N5—C33—C32	0.4 (4)
N1—Ru1—N2—C10	179.6 (2)	C33—N5—C29—C28	175.4 (2)
N2—Ru1—N1—C1	178.3 (2)	C33—N5—C29—C30	−2.2 (4)
N2—Ru1—N1—C5	−1.20 (18)	N1—C1—C2—C3	1.2 (5)
N1—Ru1—N3—C11	2.8 (3)	C1—C2—C3—C4	0.8 (5)
N1—Ru1—N3—C15	−176.6 (2)	C2—C3—C4—C5	−0.9 (4)
N3—Ru1—N1—C1	177.0 (2)	C3—C4—C5—N1	−1.0 (4)
N3—Ru1—N1—C5	−2.5 (3)	C3—C4—C5—C6	179.9 (2)
N1—Ru1—N4—C24	78.3 (2)	N1—C5—C6—N2	−1.2 (3)
N1—Ru1—N4—C28	−101.35 (18)	N1—C5—C6—C7	175.9 (2)
N4—Ru1—N1—C1	81.4 (2)	C4—C5—C6—N2	177.9 (2)
N4—Ru1—N1—C5	−98.16 (19)	C4—C5—C6—C7	−5.0 (4)
N1—Ru1—N5—C29	92.92 (19)	N2—C6—C7—C8	−2.0 (4)
N1—Ru1—N5—C33	−85.4 (2)	C5—C6—C7—C8	−178.9 (2)
N5—Ru1—N1—C1	2.6 (2)	C6—C7—C8—C9	−0.4 (4)
N5—Ru1—N1—C5	−176.89 (19)	C6—C7—C8—C16	176.4 (2)
N2—Ru1—N3—C11	1.47 (18)	C7—C8—C9—C10	1.0 (4)
N2—Ru1—N3—C15	−177.9 (2)	C7—C8—C16—C17	48.6 (4)
N3—Ru1—N2—C6	−179.91 (18)	C7—C8—C16—C21	−131.9 (3)
N3—Ru1—N2—C10	−0.9 (2)	C9—C8—C16—C17	−134.7 (3)
N2—Ru1—N4—C24	−1.5 (2)	C9—C8—C16—C21	44.8 (3)
N2—Ru1—N4—C28	178.82 (18)	C16—C8—C9—C10	−175.8 (2)
N4—Ru1—N2—C6	90.8 (2)	C8—C9—C10—N2	0.7 (4)
N4—Ru1—N2—C10	−90.1 (2)	C8—C9—C10—C11	178.0 (2)
N3—Ru1—N4—C24	−81.2 (2)	N2—C10—C11—N3	1.1 (3)
N3—Ru1—N4—C28	99.14 (18)	N2—C10—C11—C12	−179.7 (2)
N4—Ru1—N3—C11	98.61 (18)	C9—C10—C11—N3	−176.4 (2)
N4—Ru1—N3—C15	−80.8 (2)	C9—C10—C11—C12	2.8 (4)
N3—Ru1—N5—C29	−85.07 (19)	N3—C11—C12—C13	1.8 (4)
N3—Ru1—N5—C33	96.6 (2)	C10—C11—C12—C13	−177.3 (2)
N5—Ru1—N3—C11	177.11 (18)	C11—C12—C13—C14	−0.8 (4)
N5—Ru1—N3—C15	−2.3 (2)	C12—C13—C14—C15	−0.4 (5)
N4—Ru1—N5—C29	3.12 (18)	C13—C14—C15—N3	0.6 (5)
N4—Ru1—N5—C33	−175.2 (2)	C8—C16—C17—O1	−0.0 (3)
N5—Ru1—N4—C24	177.0 (2)	C8—C16—C17—C18	177.8 (3)
N5—Ru1—N4—C28	−2.62 (17)	C8—C16—C21—C20	−179.4 (2)
C22—O1—C17—C16	−168.8 (3)	C17—C16—C21—C20	0.2 (4)
C22—O1—C17—C18	13.5 (5)	C21—C16—C17—O1	−179.6 (2)
C23—O2—C20—C19	−7.0 (5)	C21—C16—C17—C18	−1.7 (4)
C23—O2—C20—C21	171.2 (3)	O1—C17—C18—C19	179.4 (3)
Ru1—N1—C1—C2	177.5 (2)	C16—C17—C18—C19	1.8 (5)
Ru1—N1—C5—C4	−177.5 (2)	C17—C18—C19—C20	−0.2 (5)
Ru1—N1—C5—C6	1.6 (2)	C18—C19—C20—O2	176.8 (3)
C1—N1—C5—C4	2.9 (4)	C18—C19—C20—C21	−1.3 (5)
C1—N1—C5—C6	−178.0 (2)	O2—C20—C21—C16	−177.0 (2)
C5—N1—C1—C2	−3.0 (4)	C19—C20—C21—C16	1.4 (4)
Ru1—N2—C6—C5	0.2 (2)	N4—C24—C25—C26	0.0 (3)
Ru1—N2—C6—C7	−177.1 (2)	C24—C25—C26—C27	1.3 (5)
Ru1—N2—C10—C9	177.8 (2)	C25—C26—C27—C28	−1.3 (5)
Ru1—N2—C10—C11	0.2 (2)	C26—C27—C28—N4	−0.0 (4)
C6—N2—C10—C9	−3.1 (4)	C26—C27—C28—C29	179.2 (3)
C6—N2—C10—C11	179.2 (2)	N4—C28—C29—N5	0.9 (3)
C10—N2—C6—C5	−178.9 (2)	N4—C28—C29—C30	178.4 (2)
C10—N2—C6—C7	3.8 (3)	C27—C28—C29—N5	−178.3 (2)
Ru1—N3—C11—C10	−1.8 (2)	C27—C28—C29—C30	−0.8 (4)
Ru1—N3—C11—C12	179.0 (2)	N5—C29—C30—C31	2.0 (4)
Ru1—N3—C15—C14	179.7 (2)	C28—C29—C30—C31	−175.4 (3)
C11—N3—C15—C14	0.3 (4)	C29—C30—C31—C32	0.2 (4)
C15—N3—C11—C10	177.7 (2)	C30—C31—C32—C33	−1.9 (5)
C15—N3—C11—C12	−1.5 (4)	C31—C32—C33—N5	1.7 (4)
Ru1—N4—C24—C25	179.0 (2)		
